# Osteoporotic mid-thoracic vertebral body fractures: what are the differences compared to fractures of the lumbar spine?—a systematic review

**DOI:** 10.1007/s00068-021-01792-z

**Published:** 2021-09-29

**Authors:** Ulrich Josef Spiegl, Max Joseph Scheyerer, Georg Osterhoff, Sebastian Grüninger, Klaus John Schnake

**Affiliations:** 1 Department of Orthopaedics, Trauma Surgery and Plastic Surgery, University of Leipzig, Liebigstr. 20, 04103 Leipzig, Germany; 2 Department of Orthopedics and Trauma Surgery, University Hospital of Cologne, Cologne, Germany; 3 Department of Orthopedics and Traumatology, Paracelsus Private Medical University Nuremberg, Nuremberg, Germany; 4 Center for Spinal and Scoliosis Surgery, Malteser Waldkrankenhaus St. Marien, Erlangen, Germany

**Keywords:** Mid-thoracic spine fractures, Osteoporotich vertebral body fractures, Frailty, Bone mineral density, Lumbar spine fractures

## Abstract

**Purpose:**

The aim of this systematically review is to detect differences between fractures located at the mid-thoracic spine compared to fractures of the thoracolumbar junction (TLJ) and the lumbar spine in osteoporotic vertebral body fractures.

**Methods:**

This review is based on articles retrieved by a systematic search in the PubMed and Web of Science database for publications regarding osteoporotic fractures of the thoracolumbar spine with respect to the fracture location. Differences in prevalence, cause of fracture, fracture healing, and outcomes between the mid-thoracic spine and the TLJ and the lumbar spine were considered.

**Results:**

Altogether, 238 articles could be retrieved from the literature search. A total of 222 articles were excluded. Thus, 16 remaining original articles were included in this systematic review comprising the topics prevalence, bone mineral density and regional blood flow, biomechanics, subsequent fractures, and outcome, respectively. The overall level of evidence of the vast majority of studies was moderate to low.

**Conclusion:**

Several differences between osteoporotic fractures of the mid-thoracic spine compared to the TLJ and the lumbar spine could be identified. Thereby, osteoporotic mid-thoracic fractures seem to be particularly more related to frailty without a history of traumatic injury compared to osteoporotic fractures of the TLJ and the lumbar spine. Additionally, the presence of severe mid-thoracic fractures predicts subsequent fractures of the hip. In contrast, subsequent fractures of the spine are less likely.

## Introduction

The anatomy and biomechanics of the mid-thoracic spine differ from the thoracolumbar junction (TLJ) and the lumbar spine: First, the orientation of the facet joints is different allowing rotational motion [27]. Next, the thoracic cage, defined as the fourth column by several authors, stabilizes the thoracic spine and leads to a higher stiffness [23]. Last but not least, the sagittal alignment of the thoracic spine consisting of a kyphosis differs tremendously from the TLJ and the lordotic lumbar spine [19].

These differences may influence frequency and the occurrence of osteoporotic vertebral fractures by low-energy trauma versus without any memorable trauma and contribute to differences in the further course of the disease with respect of outcome and complications. In this context, several studies reported of differences between osteoporotic thoracic and lumbar fractures. For example, Suzuki et al. [21] found inferior results in patients with thoracic osteoporotic fractures with gradual deterioration of the symptoms three months after non-operative treatment. Thus, it might be necessary to adapt the therapy strategy to the fracture location. So far, this has not been sufficiently analyzed. To analyze the existing evidence, a first step is to perform a systematic review including all articles dealing with osteoporotic thoracolumbar fractures that analyzed differences in between osteoporotic thoracic fractures, vertebral body fractures at the TLJ, and fractures at the lumbar vertebral spine.

The aim of this systematic review was to detect differences between fractures located at the mid-thoracic spine compared to fractures of the TLJ and the lumbar spine with regard to fracture prevalence, fracture development, and the course of fracture healing including the rate of subsequent fractures and the outcome.

## Methods

The literature search included osteoporotic vertebral body fractures of the thoracic and lumbar spine diagnosed radiologically by X-ray, computed tomography, and/or magnet resonance tomography (MRI).

A systematic search of the literature included all articles between January 1999 and May 2020. The two databases PubMed and Web of Science Core Collection were considered and searched. Excluded were articles dealing with non-osteoporotic fractures, cervical fractures, self-reporting fracture evaluation, and articles that did not present any data to differentiate between thoracic and lumbar fracture location. Additionally, case reports, reviews, and animal studies were excluded. Since data collection had already been completed at the time of PROSPERO registration, this review could not be registered with PROSPERO. Using the PICO scheme [11], the following review questions were defined:

- Are there any differences between osteoporotic fractures at the thoracic spine compared to the lumbar spine regarding prevalence, biology [bone mineral density (BMD) and blood flow], biomechanics, risk of subsequent fractures, and outcome, respectively?

The following search terms were used: (“vertebral body fracture” OR “vertebral fracture” OR “spine fracture” OR “lumbar spine fracture” OR “thoracic spine fracture” OR “thoracolumbar fracture”) AND (“osteoporosis” OR “osteoporotic” OR “insufficiency fracture” OR “elderly” OR “geriatric patients”) AND {[“English”(language)] OR [“german”(language)]} AND (“thoracic spine” OR “thoracic vertebrae” OR “thoracic vertebral body” OR “thoracal spine” OR “thoracal vertebrae”) NOT “case reports” NOT “review” NOT “cervical” NOT “sacral” NOT “odontoid”.

Subsequently, all relevant original articles were analyzed based on their levels of evidence and their appropriate conclusions. The following topic areas were defined as follows:

- Prevalence
- BMD and regional blood flow
- Biomechanics
- Subsequent fractures
- Outcome

## Results

**Fig. 1:**
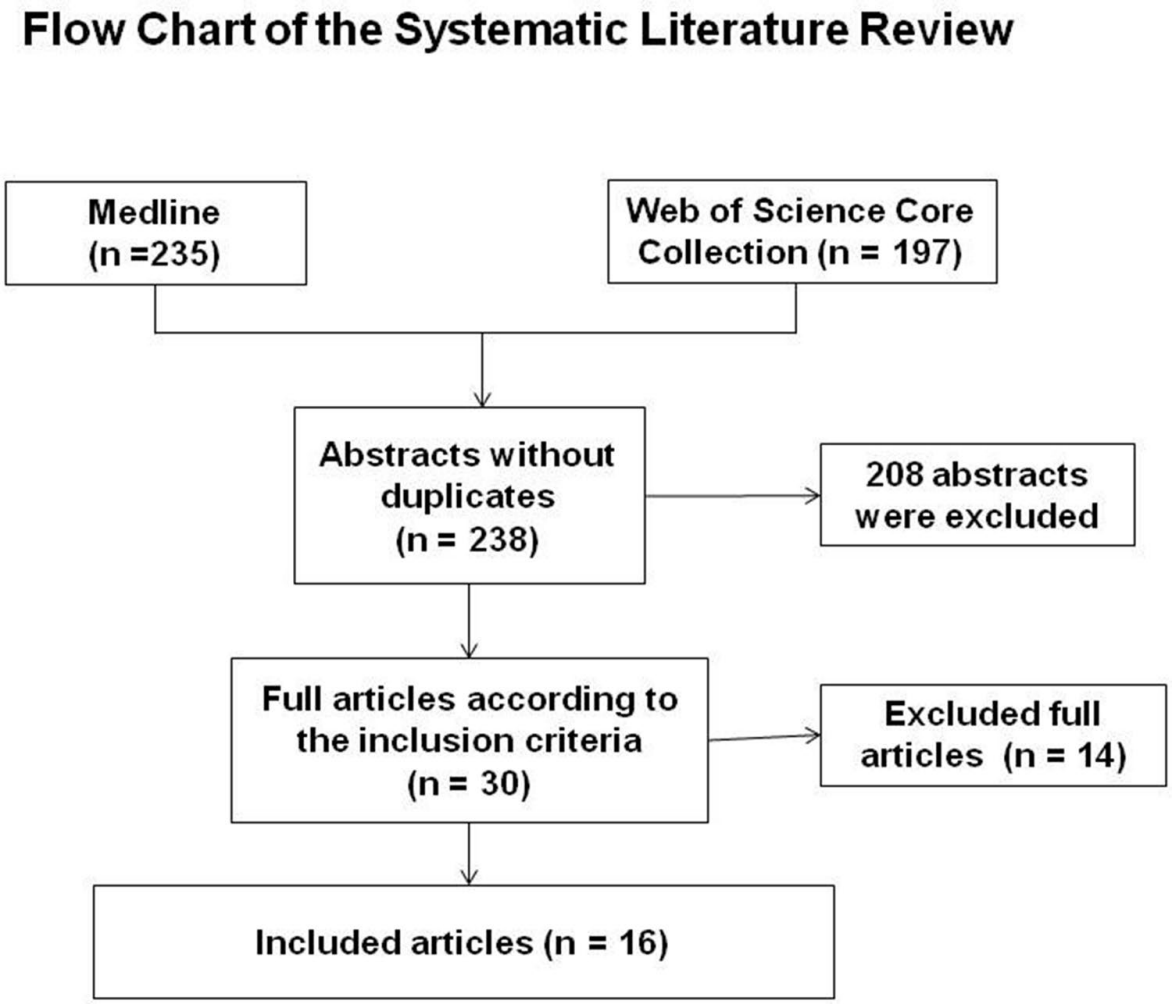
Flowchart of the systematic literature review

Altogether, 238 abstracts were retrieved from the literature search (Fig. 1). Of these, 208 articles were excluded based on abstract or title. Most of the excluded studies were overlaps between the searched literature databases, animal studies, no original articles, or were articles investigating other pathologies or studies including cervical factures or thoracic or lumbar fractures only. Altogether, 30 articles were analyzed completely. Of these articles, 14 were excluded either because not comparing the thoracic spine with the lumbar spine, not focusing on osteoporotic fractures, or no radiological fracture evaluation was performed. Altogether, 222 articles were excluded (Fig. 1). All 16 remaining original articles, which covered the period from 1999 to 2018 are summarized in Tables 1–5. Levels of evidence were defined as described by Bassler and Antes [3] (Tables 1–5).

**Table 1:** Studies dealing with the prevalence of osteoporotic thoracolumbar fractures

References	Purpose	Study design	*N*	Main message	Ev- L
Nevitt et al. [16]	Association of the number of prior vertebral fractures with the risk of new fractures and the influence of spinal location of fracture	Prospective cohort study	6082	There is a peak of osteoporotic fractures at the mid-thoracic spine and the TLJ Osteoporosis is a strong risk factor for new fractures particularly in the mid-thoracic spine	II
Waterloo et al. [24]	Age- and sex specific occurrence of osteoporotic vertebral fractures in Norway	Prospective cohort study	2887	Similar fracture distribution between the genders Majority of fractures at Th 7/8/9 and the TLJ	II

**Table 2:** Studies dealing with bone mineral density and regional blood flow

References	Purpose	Study design	*N*	Main message	Ev- L
Anderson et al. [2]	Examination how spinal location affects the relationships between quantitative computed tomography-based bone measurements and prevalent vertebral fractures	Case–control study	40/80	Vertebral fracture etiology may vary by region, with vertebral fractures in the mid-thoracic spine more strongly related to skeletal fragility	III
Watt et Crilly [26]	Association between bone mineral density T-scores and vertebral fracture location was assessed	Case–control study	120	A fracture in the mid-thoracic spine decreased the odds of having a history of traumatic injury Vertebral fractures in the lower thoracolumbar spine are associated with higher T-scores	III
Biffar et al. [4]	To evaluate contrast–enhanced MRI in vertebral bone of fractured osteoporotic vertebral bodies	Case study	10	Mean perfusion was significantly decreased in lumbar compared to thoracic vertebrae Significant perfusion alterations were observed in acute osteoporotic vertebral fractures	IV

**Table 3:** Studies dealing with biomechanics

References	Purpose	Study design	*N*	Main message	Ev- L
Bruno et al. [5]	The locations of vertebral fracture may be explained by the pattern of spinal loading	Female musculo-skeletal model	n.a	The highest factor-of-risk values generally occurred in the thoracolumbar region of the spine because these vertebrae had lower compressive strength than vertebrae in the lumbar spine	n.a
Buckley et al. [6]	To assess differences in vertebral strength between loading modes and across spinal levels	Cadaver study	30 pairs	Compressive strength was higher than flexion strength Bone mineral density and mechanics of solids values were only moderately correlated across spinal levels	n.a
Bürklein et al. [7]	Estimates of failure loads in the thoracic spine by lumbar dual energy X-ray absorptiometry are compromised of skeletal heterogeneity throughout the spine	Cadaver study	119	The correlation between thoracic failure loads (Th6 vs. Th10) was significantly higher than those between thoracic and lumbar vertebrae	
Okamoto et al. [17]	Estimate the biomechanical stress of a kyphotic deformity, with an initial vertebral fracture, place on adjacent vertebrae	Three-dimensional finite element	n.a	Existence of an initial vertebral fracture at Th12 caused an increase in stress on adjacent vertebrae and a bimodal peak in mid-thoracic vertebrae	n.a
Ignasiak et al. [13]	The effects of age-related changes in spine kinematics on thoracolumbar spinal segmental loading during dynamic activities of daily living were investigated	Case–control	44	Maximum loads predicted for the lower thoracic levels (Th9/Th10-L1/L2) were similar compared to the young Maximum compressive loads predicted for the elderly motion patterns were lower than those of the young for upper thoracic levels during stand-to-sit (Th1/Th2-Th8/Th9) and sit-to-stand (Th3/Th4-Th6/Th7)	III

**Table 4:** Studies dealing with subsequent fractures

References	Purpose	Study design	*N*	Main Message	Ev- L
Puisto et al. [18]	Evaluating the associations between the severity of vertebral fractures and the risk of subsequent hip fracture	Case–control register study	7095	The presence of a severe vertebral fracture in the thoracic spine strongly predicts subsequent hip fracture	III
Watt et al. [25]	Exploring the distribution of vertebral fractures in patients with proximal femur fractures	Retrospective cohort study	120	The distribution of vertebral fractures among patients with subcapital fractures differed from the other fracture groups Subcapital fractures and some lumbar fractures have a different underlying etiology than intertrochanteric fractures and thoracic (Th4–Th10) fractures	IV
Lenski et al. [15]	Investigating the incidence of concomitant acute osteoporotic vertebral body fractures and previous vertebral body fractures and to identify risk factors for concomitant acute vertebral body fractures	Prospective epidemiological study	1005	Patients with previous fractures of the thoracic spine between Th1 and Th9 are less likely to suffer subsequent fractures of the spine	II
Karlsson et al. [14]	Evaluates if prevalent vertebral fractures and fracture characteristics predict subsequent fractures	Prospective observational study	3014	Old men with fractures in both the thoracic and lumbar regions, and a degree of vertebral body compression in the three worst quartiles are at an especially high risk of sustaining new fractures	III

**Table 5:** Studies dealing with outcome of osteoporotic thoracolumbar fractures

Study (year)	Purpose	Study design	*N*	Main Message	Ev- L
Fechtenbaum et al. [12]	To assess the quality of life in osteoporotic postmenopausal women, according to the number and the severity of the vertebral fractures	Cohort study	629	•There was no difference in quality of life according to the thoracic or lumbar location of the fractures	III
Suzuki et al. [21]	Is the outcome after an acute osteoporotic vertebral body fracture related to the fracture level, type of fracture, and grade of fracture deformation?	Prospective case study	107	•Lumbar fractures tended to improve steadily while thoracic fractures deteriorated	III

### Prevalence

A total of two studies analyzed the frequency of fractures with respect to fracture location (Table 1). Both studies reported a peak of fractures at the TLJ and the mid-thoracic spine. Nevitt et al. [16] reported 7% of the fractures at Th12 and L1 and 5% at Th7 and Th8. Waterloo et al. [24] compared the fracture location between the genders and found a majority of fractures at Th7, Th9, Th12, and L1 in women with a similar distribution in men except of a higher frequency in Th8 instead of Th9. The highest vertebral deformities were seen in the mid-thoracic region and the TLJ.

### Bone mineral density and regional blood flow

Three papers evaluated the differences of bone mineral density (BMD) and regional blood flow between the thoracic and lumbar spine (Table 2). With the intention to predict risk of vertebral fractures, previous investigations typically measured BMD in the lumbar spine, especially in L1. Anderson et al. [2] dealt with the question to what extent these BMD values can also be used to predict thoracic fractures. Therefore, the authors performed a community-based case–control study including 40 patients (46 vertebral fractures) with and 80 patients without vertebral fractures. BMD was measured by quantitative computed tomography-based bone measures. Low BMD measured in L3 was significantly associated with a higher fracture risk at the mid-thoracic spine and the TLJ. Similar, the observations in Th10—however, the expressiveness was lower compared to L3. The relationship between low BMD in Th10 and risk of fracture lost significance at the lumbar spine. In fact, strength and factor-of-risk measurement at L3 were more strongly associated with mid-thoracic fractures than measurements at T10. Beside this, the authors concluded that vertebral fracture etiology may vary by region, with vertebral fractures in the mid-thoracic spine more strongly relating to skeletal fragility.

Similar results were found by Watt and Crilly [26]. They included 120 patients in their case–control study to determine if there is an association between vertebral fracture location and measured BMD T-score. They demonstrated that a lower lumbar BMD T-score was associated with a Th4–Th10 (*p* = 0.02) as well as the Th11–L4 vertebral fracture location (*p* < 0.001) in unadjusted analyses. After multivariable regression analyses, only the Th11–L4 fracture location remained significantly predictive of a lower lumbar BMD T-score (*p* = 0.005). The authors also examined the relationship between fracture and history of any traumatic injury depending on fracture location. They concluded that patients with vertebral fractures in the mid-thoracic spine (Th4–Th10) may be less likely to report about a traumatic cause of their vertebral fracture compared to lumbar one.

In contrast, Biffar et al. [4] examined plasma flow (PF), plasma volume (PV), and extraction flow (EF) in fractured and normal-appearing vertebrae by Dynamic Contrast-Enhanced MRI and their influence on manifestation of osteoporosis and vertebral fractures. Perfusion parameters were decreased significantly in normal-appearing vertebral bone marrow (vBM) of patients with osteoporosis compared to healthy subjects. Furthermore, significant perfusion alterations were observed in acute osteoporotic vertebral fractures compared to normal-appearing vertebrae. Interestingly, perfusion shows reproducible alterations in vBM, depending on the anatomic level. PF and PV values measured separately showed a gradual decline from Th8 to L5, indicating that lumbar vertebrae are less perfused. However, despite lower perfusion rates and lower BMD, the risk of osteoporotic fractures in the lower lumbar spine was not increased.

### Biomechanics

Five of the included studies examined biomechanical aspects (Table 3). Bruno et al. [5] analyzed spinal loading during daily activities to explain the fracture pattern and fracture distribution in a biomechanical model. The authors described the highest fracture risk at the TLJ due to a lower predicted strength compared to the lower lumbar spine. Interestingly, none of the 119 activities that were examined produced peaks in the factor-of-risk at the mid-thoracic region. Buckley et al. [6] tested the relative strength of isolated vertebral bodies under flexion and extension and reported an approximately 40% lower vertebral body strength under bending loads than pure compression. Bürklein et al. [7] studied the mechanical failure loads of thoracic and lumbar vertebrae and reported of significant lower failure loads at Th6 compared to Th10 and L3 without any significant differences of the failure loads between Th10 and L3. Okamoto et al. [17] analyzed the effect of a kyphotic deformity of 10° and 20° at Th12. This caused an increase in stress on adjacent vertebrae. Additionally, a bimodal peak of the stress was seen including the mid-thoracic region. Ignasiak et al. [13] evaluated the spinal loading effects between young and elderly individuals. Thereby, the maximum compressive loads in elderly were lower than those in young lumbar levels during flexion and for upper thoracic levels during stand-to-sit (Th1/Th2-Th8/Th9) and sit-to-stand (Th3/Th4-Th6/Th7). However, the maximum loads predicted for the lower thoracic levels (Th9/Th10–L1/L2) were similar compared to the young.

### Subsequent fractures

Four studies evaluated the risk of subsequent vertebral and/or extra-vertebral fractures after suffering an osteoporotic thoracolumbar fracture (Table 4). Generally, both can be considered as an indicator for impaired bone quality and a sign of frailty.

It could be shown that a severe thoracic vertebral fracture is a strong predictor for sustaining a subsequent hip fracture, whereas mild or moderate fractures and the number of compressed vertebrae were not found to be statistically significant risk factors [18]. In a retrospective analysis on the relation of hip fractures and concomitant vertebral fractures, patients with a femoral neck fracture were observed to differ from patients with intertrochanteric fractures or only vertebral fractures in terms of both the number and distribution of their vertebral fractures—these being fewer, frequently single, and more often confined to the lower spine [25].

Data from the Registry of Pathological Osteoporotic Vertebral Fractures (REPAPORA) with 1005 patients and 2874 osteoporotic vertebral fractures indicated that patients with previous fractures of the thoracic spine between Th1 and Th9 are less likely to suffer subsequent fractures of the spine [15]. Patients with a thoracic osteoporotic vertebral compression fracture will most likely sustain a subsequent fracture of the TLJ followed by the mid-thoracic spine while patients with an osteoporotic fracture of the lumbar spine will most likely have a subsequent fracture at the TLJ or the lumbar spine [15]. The authors of this registry study named this phenomenon of a higher likelihood for subsequent fractures in the vertebral segments below the index fracture “lumbar drift” and conjectured that this may be due to kyphotic deformity and increased spinal load in the more caudal vertebral bodies.

This could also be the mechanics behind the clinical observation that elderly men with fractures in both the thoracic and lumbar regions are at an especially high risk of sustaining secondary fractures [14].

### Outcome

Two studies evaluated the clinical outcome of patients suffering of osteoporotic thoracolumbar fractures and analyzed the impact of fracture location (Table 5). Fechtenbaum et al. [12] included 629 post-menopausal women and reported no differences in the clinical outcome between thoracic and lumbar vertebral fractures location. In contrast, the authors found a significant association between both grades of vertebral deformity and number of fractures with inferior outcomes. In contrast, Suzuki et al. [21] evaluated 107 geriatric patients with thoracolumbar fractures and found that patients with lumbar fractures tended to improve steadily, whereas those with thoracic fractures tended to deteriorate after the improvement of the first three months. The authors postulated that the deterioration might be caused by the increased kyphosis at the thoracic spine leading to muscular overstrain.

## Discussion

The most important findings of this study are the identification of differences between osteoporotic fractures of the mid-thoracic spine compared to the TLJ and the lumbar spine. Mid-thoracic fractures seem to be more related to frailty depicted in several of the above mentioned studies. This can explain the inferior results that were seen after mid-thoracic vertebral fractures with deterioration after the first three months. In contrast, Fechtenbaum et al. [12] described no differences in the quality of life between osteoporotic thoracic and lumbar fractures. However, the authors evaluated the quality of life based on deformities on radiographs that were interpreted as a fracture and concomitant pain in this region. Thereby, it was not possible to differentiate between fresh and old fracture situations and estimate the influence of back pain due to degenerative pathologies leading to a decrease in accuracy of their conclusions. Notwithstanding, the authors found an association between severity of deformity and inferior results. This is in accordance with the literature [8, 10] and can be explained by the high bending moments acting on the mid-thoracic spine during normal activities of daily living [1, 9]. These loads acting on the vertebrae of reduced bone quality might contribute to a higher fracture risk for the elderly [13]. Interestingly, a deformity of Th12 led to a bimodal stress peak at the adjacent vertebral bodies as well at Th 7 and Th 8 [17]. Thus, a higher grade of deformity and inferior results can be explained. However, no increased osteoporotic fracture rate was noticed at the mid-thoracic spine compared to other regions of the spine. The rate of osteoporotic fractures is similar in the mid-thoracic spine and the TLJ [16, 24]. This can be partly explained by the higher BMDs and better perfusion at the mid-thoracic spine compared to the TLJ [2, 4]. Additionally, the thoracic spine is stabilized by the rib cage leading to more stiffness [7, 23]. Thus, fractures of the thoracic spine might rather occur in patients suffering from frailty [2, 20].

Unfortunately, there were no studies dealing with differences of fracture healing between fractures of the thoracic and lumbar vertebral spine. Similarly, no evidence exists regarding our hypothesis that it might be necessary to adapt the treatment strategies with respect of fracture location what we have to leave unanswered.

With respect to the rate of subsequent fractures, a high rate of adjacent and subsequent fractures of the spine might be expected. A meta-analysis published in 2017 reported an overall adjacent fracture rate of 20% within one year after the initial fracture—regardless whether the fracture was treated operatively or non-operatively [28]. Similar numbers were observed by a recent study that looked at symptomatic adjacent fractures after osteoporotic vertebral compression fractures of the thoracolumbar spine [22]. In their study, the authors could not detect any association between index fracture level and the occurrence of adjacent fractures. In contrast, Lenski et al. [15] stated that patients with prevalent fractures of the mid-thoracic spine were less likely to suffer from subsequent fractures of the spine. Additionally, Puisto et al. [18] found a strong prediction between severe osteoporotic mid-thoracic vertebral body fractures and subsequent hip fractures. This might be caused by a different fracture etiology causing mid-thoracic fractures as described by Watt et al. [25].

Generally, this study has several limitations. The search strategy might have missed articles by the used search items and selectively including articles dealing with the thoracic spine only. The level of evidence in the majority of studies was low, leading to a limited conclusion that can be drawn out of it. The results of some of the studies were not totally consistent. This might be caused by the differences in the study populations, differences in the methodology of fracture detection, and differences in the treatment strategies. Last but not least, the high number of studies with low evidence level was the reason to present the results in a narrative manner without any statistical evaluation of the strength of evidence and the precision of outcome parameters.

Altogether, further studies are necessary to identify and quantify differences of the location of osteoporotic vertebral body fractures between the mid-thoracic and the lumbar spine. Thereby, potential consequences on diagnostic and treatment strategies for the mid-thoracic spine would be of greatest interest.

## Conclusion

Several differences between osteoporotic fractures of the mid-thoracic spine compared to the TLJ and the lumbar spine could be identified. Thereby, osteoporotic mid-thoracic fractures seem to be particularly more related to frailty without a history of traumatic injury compared to osteoporotic fractures of the TLJ and the lumbar spine. Additionally, the presence of severe mid-thoracic fractures predicts subsequent fractures of the hip. In contrast, subsequent fractures of the spine are less likely.
